# Deficits in neuromuscular control of increasing force in patients with chronic lateral epicondylitis

**DOI:** 10.3389/fphys.2023.1178557

**Published:** 2023-08-11

**Authors:** Yueh Chen, Chia-Ling Hu, Chih-Kai Hong, Kai-Lan Hsu, Fa-Chuan Kuan, Wei-Li Chen, Wei-Ren Su, Yi-Ching Chen, Ing-Shiou Hwang

**Affiliations:** ^1^ Institute of Allied Health Sciences, College of Medicine, National Cheng-Kung University, Tainan, Taiwan; ^2^ Department of Orthopedics, Madou Sin-Lâu Hospital, Tainan, Taiwan; ^3^ Department of Orthopaedic Surgery, National Cheng Kung University Hospital, College of Medicine, National Cheng Kung University, Tainan, Taiwan; ^4^ Musculoskeletal Research Center, Innovation Headquarter, National Cheng Kung University, Tainan, Taiwan; ^5^ Department of Physical Therapy, College of Medical Science and Technology, Chung Shan Medical University, Taichung, Taiwan; ^6^ Department of Physical Therapy, College of Medicine, National Cheng Kung University, Tainan, Taiwan

**Keywords:** tennis elbow, motor unit, force variability, EMG, muscle strength

## Abstract

**Objective:** This study investigated the neuromuscular control of increasing and releasing force in patients with chronic lateral epicondylitis (CLE).

**Methods:** Fifteen patients with CLE (10 males, 5 females, 46.5 ± 6.3 years) and fifteen healthy participants (9 males, 6 females, 45.3 ± 2.5 years) participated in this study. In addition to power grip and maximal voluntary contraction (MVC) of wrist extension, force fluctuation dynamics and characteristics of inter-spike intervals (ISI) of motor units (MUs) with various recruitment thresholds in the extensor carpi radialis brevis (ECRB) and extensor carpi radialis longus (ECRL) during a designated force-tracking task with a trapezoidal target (0%–75%–0% MVC) were assessed.

**Results:** Besides a smaller MVC of wrist extension, the patients exhibited significantly greater task errors (*p* = 0.007) and force fluctuations (*p* = 0.001) during force increment than the healthy counterparts. Nevertheless, no force variables significantly differed between groups during force release (*p* > 0.05). During force increment, the amplitudes of the motor unit action potential of the ECRB and ECRL muscles of the patients were smaller than those of the heathy counterparts (*p* < 0.001). The patient group also exhibited a higher percentage of motor units (MU) with lower recruitment threshold (<5% MVC) in the ECRL/ECRB muscles and a lower percentage of MU with higher recruitment threshold (>40% MVC) in the ECRB muscle, compared to the healthy group. During force increment, the patient group exhibited a higher rate of decrease in inter-spike intervals (ISIs) of motor units with lower recruitment thresholds (<10% MVC) in the ECRB and ECRL muscles, compared to the control group (*p* < 0.005).

**Conclusion:** The patients with CLE exhibited more pronounced impairment in increasing force than in releasing force. This impairment in increasing force is attributed to deficits in tendon structure and degenerative changes in the larger motor units of the wrist extensors. To compensate for the neuromuscular deficits, the rate of progressive increase in discharge rate of the remaining smaller motor units (MUs) is enhanced to generate force.

**Significance:** The deficits in neuromuscular control observed in CLE with degenerative changes cannot be fully explained by the experimental pain model, which predicts pain-related inhibition on low-threshold motor units.

## 1 Introduction

Lateral epicondylitis (LE), also known as tennis elbow, impairs the functioning of the common wrist extensors, especially the extensor carpi radialis brevis (ECRB), in athletes, computer users, carpenters, and so on. LE occurs in 1%–3% of the general population in the fifth decade of life due to repetitive and vigorous overuse of the common wrist extensors (Thurston, 1998; Shiri et al., 2006). Mechanically, eccentric contraction of the wrist extensors leads to microscopic tendon tears, which evolve into necrotic muscle fibers (Ljung et al., 1999) and degenerative tendinosis of the ECRB tendon (Kraushaar and Nirschl, 1999; Nirschl and Ashman, 2003; Tosti et al., 2013) in 91% of patients with LE (Nirschl and Petterone, 1979). Pain frequently develops during imposed stretch when the wrist extensors contract against a heavy load. The force generation capacity of patients with LE may be weakened (Sesto et al., 2006), as indexed with declines in peak grip power and maximal voluntary contraction (MVC) of wrist extension (Bisset et al., 2006; Andersen et al., 2008; Christou, 2011). Despite a decrease in maximal force, little attention has been paid to force precision control (or force gradation) in patients with LE. Impairment of precise control in increasing or releasing force is related to daily activities such as lifting and holding of low-mass objects as well as instrumented object usage. As force increment and force release are differentially regulated by the central nervous system (Park et al., 2016), young and older adults consistently demonstrate poorer task performance with greater force fluctuations during force release than during force increase (Erim et al., 1999; Cogliati et al., 2019). Theories on the motor adaptation to pain also posit that movement solutions, such as force production and movement velocity, are tuned to minimize excitation of peripheral nociceptors (Lund et al., 1991; Hodges and Tucker, 2011). To date, whether force increment and force release are differently affected in patients with chronic lateral epicondylitis (CLE) is not clear, given that pain and degenerative changes in the muscle–tendon system coexist for a period of time. Neural drive and motor unit activities could vary with tendinopathy due to atypical mechanical properties and load distributions in the tendons (Fernandes et al., 2022).

Surface electromyography (EMG) can be used to assess compromised neuromuscular function of the forearm muscle in patients with CLE. Previous studies have revealed compensatory increases in activation of the common wrist extensor during power grip (Heals et al., 2016) and ball stroke (Kelley et al., 1994; De Smedt et al., 2007), which involve coactivation of wrist flexors and extensors to maintain joint stiffness. On the other hand, muscle activation of the ECRB during computer work (Samani et al., 2011), submaximal grip (Alizadehkhaiyat et al., 2007; Alizadehkhaiyat et al., 2009), and low-load isometric wrist extension (Rojas et al., 2007) have been shown to be smaller in patients. The decrease in muscle activation due to LE could be attributable to pain-related inhibition (Lund et al., 1991; Tucker et al., 2009), as in many other musculoskeletal disorders. Also, the ECRB of patients with LE is susceptible to muscle fatigue, as supported by a more rapid decline in the median frequency of surface EMG during prolonged isometric contraction (Alizadehkhaiyat et al., 2007; Alizadehkhaiyat et al., 2009). To our knowledge, only Calder et al. (2008) has investigated motor unit activities of the ECRB muscle during a low-level resisted wrist extension (5%–20% MVC) in patients with LE (Calder et al., 2008). The authors reported smaller areas (surface-detected) and longer durations (needle-detected) of motor unit action potentials (MUAP) in patients than in healthy subjects. However, the comparison of motor unit morphology in the work was criticized, as the patient and healthy groups were not age matched (Heales et al., 2016). In addition, the previous work simply investigated low-level force control in a narrow range, without examination of the functioning of motor units with higher recruitment thresholds.

In addition to force generation capacity (peak power grip and MVC of wrist extension), this study aimed to investigate deficits in broad-range force gradation of wrist extension in patients with CLE, with a particular focus on task errors and force fluctuation properties during force increment and force release. Next, with decomposed surface EMG, the discharge patterns of motor units were contrasted between the patients and healthy subjects, when the group difference in task performance of force increment or force release was significant. Finally, this study characterized the functional linkages between deficits in neuromuscular control (force fluctuation properties and corresponding MU discharge patterns) and self-reported questionnaires used in clinics, as well as force generation capacity in patients with CLE.

## 2 Methods

### 2.1 Subjects

Thirty participants, including 15 patients with chronic lateral epicondylitis (CLE) (*n* = 15; 10 males, 5 females, age: 46.5 ± 6.3 years; body mass: 75.7 ± 13.1 kg; height: 168.3 ± 8.2 cm) and 15 healthy participants (*n* = 15; 10 males, 5 females, age: 45.3 ± 2.5 years; body mass: 75.1 ± 14.9 kg; height = 169.1 ± 6.1 cm) participated in this study. No participants had signs or symptoms of cervical radiculopathy and/or other repetitive strain injury (such as carpal tunnel syndrome). Besides physical examination and musculoskeletal ultrasound (MSK-US) performed by an experienced orthopedist. We utilize the Connell method for diagnosing tennis elbow (lateral epicondylitis) through ultrasound examination. Diagnosis was confirmed as focal hypoechoic area in the deep part of the ECRB tendon, found in all participants (15/15) (Connell et al., 2001). The inclusion of CLE patients was re-confirmed with symptoms of lateral epicondylitis for at least the past 3 months and pain greater than five on the Visual Analogue Scale (VAS) on the lateral epicondyle when palpated (Mishra et al., 2014). Disabilities of the arm, shoulder and hand (DASH) scores and patient-rated tennis elbow evaluation (PRTEE) scores were also recorded to index clinical symptoms and functional impairments of CLE. The demographic characteristics of the CLE and control groups are summarized in Table 1. The study was approved by the Institutional Review Board for Human Subjects of the Sin-Lau Hospital (No. SLH-110-A-001), and all subjects signed a written informed consent form before the experiment.

**TABLE 1:** Demographic characteristics of chronic lateral epicondylitis (CLE) and control groups.

*n* = 15	CLE	Control	Statistics
Height (cm)	168.3 ± 8.2	169.1 ± 6.1	*t*_*28*_ = .034, *p* = 0.913
Body weight (kg)	75.7 ± 13.1	75.1 ± 14.9	*t*_*28*_ = .115, *p* = 0.909
Age (year)	46.5 ± 6.3	45.3 ± 2.5	*t*_*28*_ = .210, *p* = 0.835
Male vs. Female	10:5	10:5	*χ* ^*2*^_(1)_ = .00, *p* = 1.000
Dominant vs. Non-dominant	11:4	11:4	*χ* ^*2*^_(1)_ = .00, *p* = 1.000
DASH score	35.1 ± 16.1	N/A	N/A
PRTEE	46.6 ± 18.2	N/A	N/A
VAS	7.1 ± 1.8	N/A	N/A

### 2.2 Experimental procedures

**FIGURE 1:**
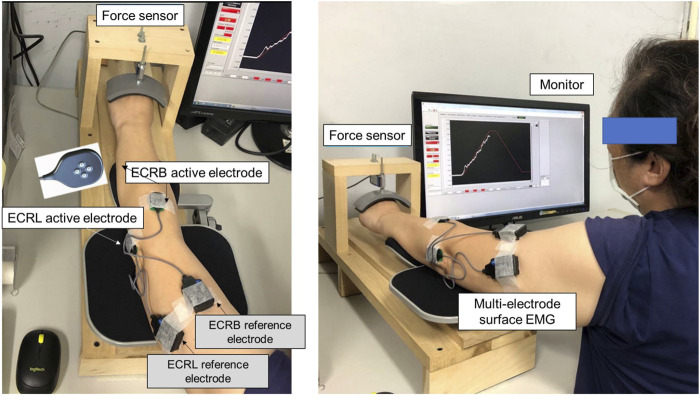
Experimental setup to measure wrist extension force and electromyographic signals from the extensor carpi radialis brevis (ECRB) and extensor carpi radialis longus (ECRL) during trapezoidal isometric contraction.

**FIGURE 2:**
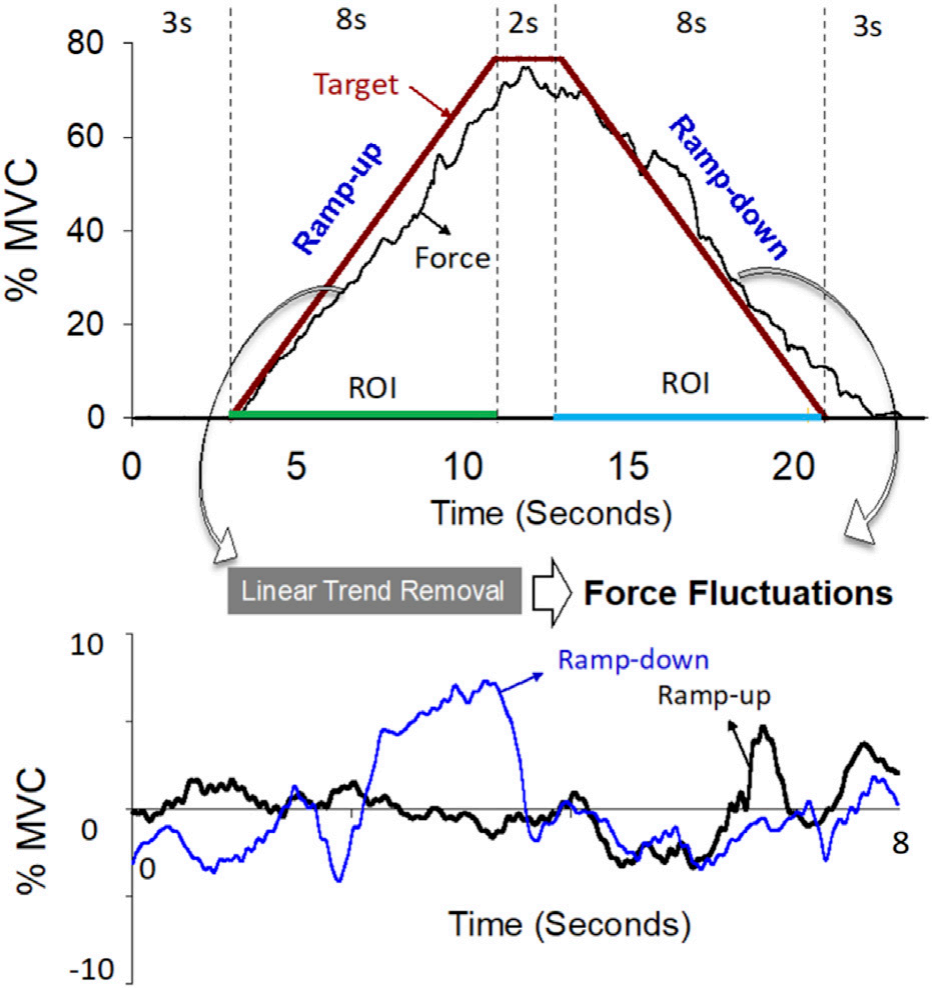
The upper plot is a representative trial of the force data and target line of the isometric trapezoidal contraction (0%–75%–0% MVC). The lower plot shows trajectories of force fluctuations in the ramp-up and ramp-down phases. Force fluctuations are defined as force outputs in the regions of interest (ROI) after removal of linear trends.

All participants in the control and CLE groups completed two strength measures (power grip and maximal voluntary contraction (MVC_WE_) for wrist extension) and force gradation task. Along with grip strength, the VAS, DASH scale, and PRTEE of patients with CLE were measured on the day of the first visit. The power grip was performed in a neutral wrist position, roughly 20° wrist flexion and 20° wrist extension. The maximal grip force (GF_max_) was determined from three separate trials interleaved with 3 minutes of rest. MVC_WE_ was first assessed on the day of the main experiment (the second visit). With appropriate space for wrist extension, the participants were seated with the palm and forearm located within a plastic splint on a wooden platform (Figure 1, left). The MVC_WE_ was the peak value of three maximum contraction trials, separated by 3-min rest periods. Then the participants in the control and patient groups performed a designated trapezoidal force-tracking task controlled by isometric wrist extension with ramp-up and a ramp-down phases. The force-tracking task was used to assess the force precision control of force increment and force release of the wrist extensors. Under visual guidance, the participants exerted the isometric force of wrist extension to couple a ramp up–hold–ramp down target signal displayed on a 24-inch computer monitor (1920 × 1,080 pixels) (Figure 1, right). The target signal for force-tracking with wrist extension consisted of a 3-s latent period and an 8-s ramp-up phase to 75% MVC, 2 s of the static level for the 75% MVC isometric force task, an 8-s ramp-down phase to rest, and 3 s of latency at the end (Figure 2, upper). Each contraction trial lasted a total of 24 s. Three force-tracking trials interleaved with 3-min rest periods were completed by all participants. The particular trapezoidal contraction was designed for EMG decomposition using a previous proof-of-algorithm (De Luca et al., 2015), which can be used to decompose surface EMG into motor unit action potential (MUAP) trains for dynamic isometric contraction up to 80% MVC. Force fluctuations of the 8-s ramp-up (4th to 11th seconds) and 8-s ramp-down (14th to 21st seconds) phases were defined as force outputs in the regions of interest after the removal of linear trends (known as detrending processing) (Figure 2, lower). The force fluctuations reflected the force scaling capacity during the ramp-up and ramp-down phases. During the force-tracking task, two 4-pin wireless surface EMG electrodes (sEMG) were used to detect the muscle activities of the extensor carpi radialis brevis (ECRB) and extensor carpi radialis longus (ECRL).

### 2.3 Instrumentation

Grip strength was evaluated with a custom-made dynamometer (Fabrication Enterprises, White Plains, NY, United States). The tracking force of isometric wrist extension was measured with a force sensor (Model: MB-100, Interface Inc., Scottsdale, AZ, United States) and then conditioned with an analog low-pass filter (cut-off frequency: 6 Hz) to exclude the high-frequency force components independent of visuo-motor processes. The force signal was sampled at 1 kHz with a NI-DAQ card (model USB6251; National Instruments, Austin, United States) controlled by a custom program on a LabVIEW platform (LabVIEW v.8.5, National Instruments Inc., Austin, TX, United States). The myoelectrical signals of the ECRL and ECRB muscles were detected with two wireless sensor arrays (Trigno Galileo sensor; Delsys Inc., Natick, MA, United States). The head of each sensor array (size: 23 × 30 × 7 mm; Mass: 19 g) houses four active electrodes in a diamond formation with an inter-electrode distance of 5 mm. Low baseline noises were carefully monitored to ensure the high quality of the sEMG signals before the formal force-tracking experiment. The sEMG signals during the force-tracking task were sampled at 2000 Hz with on-line band-pass filtering of 20–450 Hz before being streamed to EMG works v.4.7.8 software (Delsys Inc., Natick, MA, United States). Data acquisition for the EMG and force systems was synchronized with a common voltage pulse.

### 2.4 Data analysis

The force fluctuations of the ramp-up and ramp-down phases were down-sampled to 100 Hz. The size and complexity of the force fluctuations were represented with root mean square (FF_RMS_) and sample entropy (FF_SampEn_), respectively (Hwang et al., 2017; Chen et al., 2018). The mathematical formula of sample entropy was $SampEn\left(m,r,N\right)=-\log\left(\sum_{i=1}^{N-m}A_{i}/\sum_{i=1}^{N-m}B_{i}\right)$ , where *r* = 15% of the standard deviation of the data, *m* is the length of the template (*m* = 2), and *N* is the number of data points in the time series. *A*_*i*_ is the number of matches of the *i*th template of length *m + 1* data points, and *B*_*i*_ is the number of matches of the *i*th template of length *m* data points (Richman and Moorman, 2000). With a smaller SampEn, greater regularity is associated with more attentive control over force fluctuations, and *vice versa*.

**FIGURE 3:**
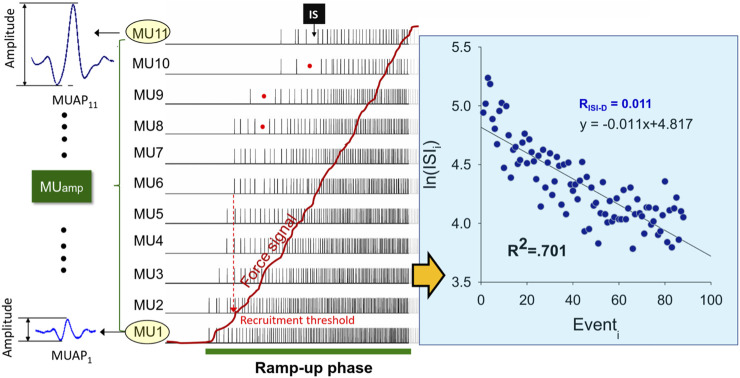
Schematic illustration of motor unit action potentials (MUAP) and discharge events of the ramp-up phase after surface decomposition. The size of a MU is indexed with peak-to-peak amplitude of the MUAP (MU_amp_). The red dots show accidental long inter-spike interval (ISI) which are discarded for further analysis according to the exclusion criteria. To analyze the progressive decrease in the time series of the ISI as force increases, a logarithmic regression model is employed in this study. The absolute value of the regression slope, denoted as R_ISI-D_, represents the rate of progressive decrease in ISI for increasing force. The coefficient of determination (R^2^) indicates the goodness of fit of the model to the ISI time series.

The software used for the post-decomposition processing of action potential morphology was NeuroMap v.1.2.1. For the whole tracking session, four-channel sEMG signals were decomposed into MUAP waveforms and discharge events using decomposition algorithms of the software (Figure 3, left) (De Luca and Contessa, 2015), which were previously validated with the decompose–synthesize–decompose–compare (DSDC) (De Luca et al., 2006; De Luca and Contessa, 2012) and two-source methods (Hu et al., 2014). In this study, only MUs with decomposition accuracy higher than 85% with the DSDC were considered for further analysis. The accuracy selection was a compromise setting between the 80% criterion set in a previous study (Madarshahian et al., 2021; Madarshahian and Latash, 2021) and the 90% criterion recommended by the manufacturer of the EMG system (Delsys) for analysis of as many MUs as possible. The inter-spike intervals (ISI) of those well-identified MUs were windowed in the periods of the ramp-up phase and ramp-down phase. The ISIs smaller than 30 ms or greater than 400 ms were empirically excluded, as they were rarely observed in the wrist extensors during submaximal contraction (Palmer and Fetz, 1985; Romaiguère et al., 1989). For the ramp-up and ramp-down phases, the progressive decrease and increase in inter-spike interval (ISI) of a motor unit were empirically modeled using the regression equation $\ln\left({ISI}_{i}\right)=m*discharge\;event\left(i\right)+k$ , where *m* represents the rates of progressive decrease and increase of the *i*-th inter-spike interval for increasing and decreasing forces, respectively (Figure 3, right). Coefficient of determination (R^2^) measured how well the model fitted the data ranging between 0 and 1. The higher the value of R^2^, the better the model is at predicting the data. In addition, the amplitude of each MU was determined from the maximal peak-to-peak values of MUAP morphology after sEMG decomposition. In terms of % MVC, the recruitment threshold of a motor unit was defined as the force intensity required to activate the motor unit for the first time (or the timing of the first discharge) during the ramp-up phase. Signal processing was completed in Matlab v.2019 (Mathworks Inc., United States).

### 2.5 Statistical analysis

The independent *t*-test was used to contrast the significance of height, weight, and age between the CLE and control groups. The chi-squared test was used to contrast the distribution of gender and limb dominance between the CLE and control groups. Multivariate Hotelling’s T^2^ test with *post hoc* analysis based on the Welch *t*-test were used to examine the differences between groups (CLE vs. control) in the strength variables (MVC_WE_ and GF_max_), performance variables of the force gradation task (task error, FF_RMS_, and FF_SampEn_) during the ramp-up and ramp-down phases. Pearson correlation was used to assess the significance of correlations between group-dependent differences in force fluctuation variables and psychometric/strength measures.

This study particularly focused on motor unit variables during the ramp-up phase, when significant group-dependent differences in force fluctuations were observed. The rate of progressive decrease in ISI (R_ISI-D_) of motor units were examined during this phase. Motor units were categorized based on their recruitment thresholds, including <10% MVC, 10%–20% MVC, and 20%–40% MVC. No motor unit with a recruitment threshold higher than 40% MVC was identified in the patient group in this study. A permutation test of performed 10,000 times was used to compare MU_amp_ of all motor units and the rate of progressive decrease in ISI (R_ISI-D_) of subpopulations of motor units with various recruitment threshold bands between the CLE and control groups. Statistical analyses were performed in the statistical package for Social Sciences (SPSS) for Windows v. 22.0 (SPSS Inc., United States).

## 3 Results

The demographic data are shown in Table 1. None of the demographic characteristics, including height, weight, age, gender, and limb dominance, were different between groups (*p* > 0.05). Table 2 contrasts the strength measurements of MVC of isometric wrist extension (MVC_WE_) and maximal grip force (GF_max_) between the CLE and healthy control groups. The result of Hotelling’s T^2^ test revealed a significant group difference in strength measures (Hotelling’s T^2^ = .349, *p* = 0.018). Post-hoc analysis indicated that the CLE group showed a smaller MVC__WE_ than the control group (201.3 ± 118.7 NT vs. 306.2 ± 107.7 NT, *p* = 0.017), rather than GF_max_ (*p* = .890) (Table 2). Table 3 contrast performance variables of the ramp-up and ramp-down phases of the force gradation task between the CLE and control groups. Task performance of the ramp-up phase of the force tracking task was significantly group-dependent (Hotelling’s T^2^ = 0.580, *p* = 0.007), unlike that of the ramp-down phase (Hotelling’s T^2^ = 0.042, *p* = 0.782). In addition to greater task errors (*p* = 0.033), *post hoc* analysis revealed that the CLE group exhibited larger RMS (FF_RMS_) (*p* = 0.001) of force fluctuations than the control group did, whereas SampEn of force fluctuations (FF_SampEn_) did not differ between groups during the ramp-up phase (*p* = .226) (Table 3). All performance variables were not group dependent during the ramp-down phase (Table 3). As the group difference in force fluctuations was evident only in the ramp-up phase, for brevity, Pearson correlation was assessed between FF_RMS_ during the ramp-up phase and psychometric/strength measures (Table 4). The FF_RMS_ during the ramp-up phase was negatively correlated to MVC_WE_ (*r* = −0.660, *p* = 0.007), despite marginally negative correlations between FF_RMS_ and GP_max_ (*r* = −0.494, *p* = 0.061)/VAS (*r* = −0.472, *p* = 0.076). In addition, FF_SampEn_ was positively correlated to MVC_WE_ (*r* = 0.562, *p* = 0.029).

**TABLE 2:** The contrast of strength variables between the chronic lateral epicondylitis (CLE) and control groups. (MVC_WE_: maximal voluntary contraction of wrist extension; GF_max_: maximal grip force) (*: CLE < Control, *p* < 0.05).

	CLE	Control	Statistics
MVC_WE_ (NT)	201.3 ± 118.7*	306.2 ± 107.7*	Hotelling’s T^2^ = 0.349, *p* = 0.018
GF_max_ (kg)	32.2 ± 11.8	32.6 ± 5.4	MVC: *t*_*28*_ = −2.534, *p* = 0.017; Grip Power: *t*_*28*_ = −0.139, *p* = 0.890

The significant statistical differences between the experimental group and the control group are indicated by the bold values.

**TABLE 3:** The contrast of performance variables between the chronic lateral epicondylitis (CLE) and control groups. (A) Ramp-up phase, (B) Ramp-down phase. (FF_RMS_: root mean square of force fluctuations, FF_SampEn_: sample entropy of force fluctuations) (^†^: CLE > Control, *p* < .05; ^††^: CLE > Control, *p* < .005).

Ramp-up	CLE	Control	Statistics
Error (%MVC)	**4.14** ± **1.71** ^**†**^	**2.77** ± **1.65**	Hotelling’s T^2^ = 0.580, *p* = 0.007
FF_RMS_ (% MVC)	**2.00** ± **0.48** ^††^	**1.46** ± **0.33**	Error: *t*_*28*_ = 2.241, *p* = 0.033; FF_RMS_: *t*_*28*_ = 3.524, *p* = 0.001
FF_SampEn_	0.15 ± 0.51	0.18 ± 0.59	FF_SampEn_: *t*_*28*_ = −1.237, *p* = 0.226
(A)			

Ramp-down	CLE	Control	Statistics
Error (%MVC)	4.46 ± 1.74	3.86 ± 1.35	Hotelling’s T^2^ = 0.042, *p* = 0.782
FF_RMS_ (% MVC)	2.06 ± 0.81	1.91 ± 0.70	Error: *t*_*28*_ = 1.058, *p* = 0.299; FF_RMS_: *t*_*28*_ = .552, *p* = 0.585
FF_SampEn_	0.17 ± 0.33	0.18 ± 0.60	FF_SampEn_: *t*_*28*_ = −0.340, *p* = .737
(B)			

The significant statistical differences between the experimental group and the control group are indicated by the bold values.

**TABLE 4:** Pearson correlations between force fluctuation variables during the ramp-up phase and strength/psychometric measures. (FF_RMS_, root mean square of force fluctuations; FF_SampEn_, sample entropy of force fluctuations) (*: *p* < .05).

Ramp-up	MVC_WE_	GP_max_	VAS	PRTEE	DASH	MSK_US
FF_RMS_	r = −0.660* (*p* = 0.007)	r = −.0494 (*p* = 0.061)	r = −0.472 (*p* = 0.076)	r = −0.206 (*p* = 0.462)	r = −0.185 (*p* = 0.507)	r = −0.202 (*p* = 0.470)
FF_SampEn_	r = 0.562* (*p* = 0.029)	r = 0.300 (*p* = 0.278)	r = 0.152 (*p* = 0.588)	r = −0.004 (*p* = 0.989)	r = −0.007 (*p* = 0.980)	r = −0.080 (*p* = 0.776)

The significant statistical differences between the experimental group and the control group are indicated by the bold values.

The numbers of MUs decomposed from multi-electrode surface electromyography (EMG) of the ECRB and ECRL from the fifteen subjects in the CLE group were 158 and 128, respectively. On average, the number of decomposed motor units in the ECRB and ECRL muscles ranged from 8–23 (11.9 ± 4.4) and 7–20 (10.9 ± 4.4), respectively. The numbers of decomposed MUs of the ECRB and ECRL from the fifteen subjects in the control group were 183 and 199, respectively. On average, the number of decomposed motor units in the ECRB and ECRL muscles ranged from 7–21 (12.5 ± 4.9) and 8–25 (13.6 ± 5.6), respectively.

**FIGURE 4:**
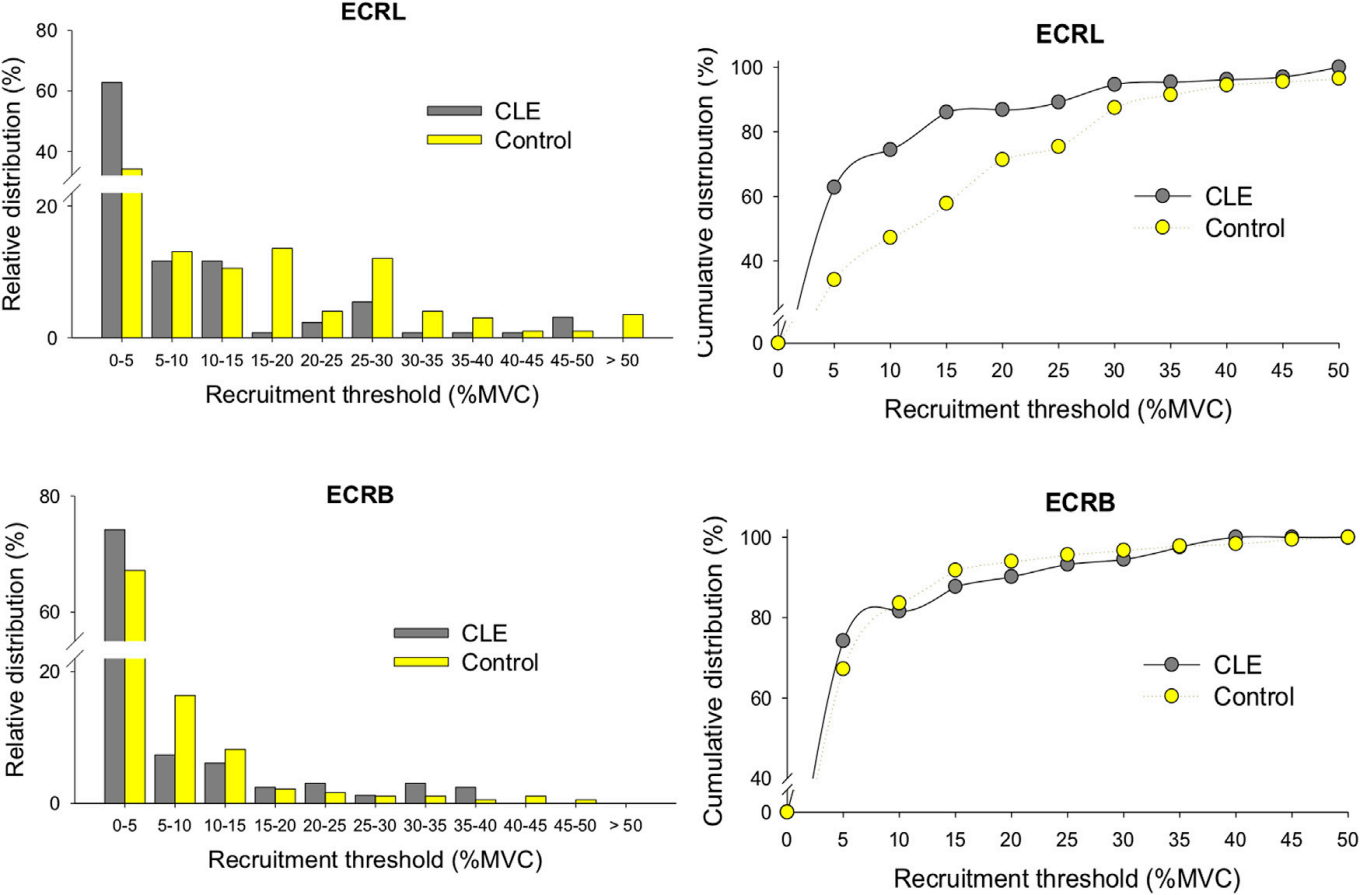
The relative distributions and cumulative distribution of recruitment thresholds of the decomposed motor units of the ECRL and ECRB muscles for the patient and control groups. (CLE, chronic lateral epicondylitis).

MU amplitude of the ECRL and ECRB muscles for increasing force were contrasted between the CLE and control groups (Table 5), as only force fluctuations during the ramp-up phase were group dependent. For the ECRL muscle, the mean amplitude of MUAP (MU_amp_) was significantly smaller in the CLE group (206.0 ± 175.6 µV) compared to the control group (302.4 ± 267.8 µV) (*p* < 0.001). Similarly, the MU_amp_ for the ECRB muscle was smaller in the CLE group (158.2 ± 103.6 µV) compared to the control group (237.9 ± 221.2 µV) (*p* < 0.001). Figure 4 displays the relative distribution and cumulative distribution of pooled motor units with various recruitment thresholds in the EDRL and ECRB muscles for the patient and control groups. The majority of the identified motor units in both the CLE and control groups exhibited low recruitment thresholds below 5% MVC. Patients with CLE had a greater percentage of low-threshold motor units than the control group. Additionally, the ECRB muscle in the patient exhibited fewer motor units with recruitment thresholds >40% MVC. The ECRL muscle in the patients generally exhibited fewer motor units with recruitment thresholds >15% MVC, except for the recruitment threshold range of 45%–50%.

**TABLE 5:** The contrast of motor unit action potential amplitude (MU_amp_) between the extensor carpi radialis brevis (ECRB) and extensor carpi radialis longus (ECRL) between the chronic lateral epicondylitis (CLE) and control groups. (***, CLE < Control, *p* < 0.001).

MU_amp_ (uV)	CLE	Control	Statistics
ECRL	206.0 ± 175.6***	**302.4 ± 267.8**	*t*_*28*_ = −3.661, *p* < 0.001
ECRB	158.2 ± 103.6***	**237.9 ± 221.2**	*t*_*28*_ = −4.233, *p* < 0.001

The significant statistical differences between the experimental group and the control group are indicated by the bold values.

**FIGURE 5:**
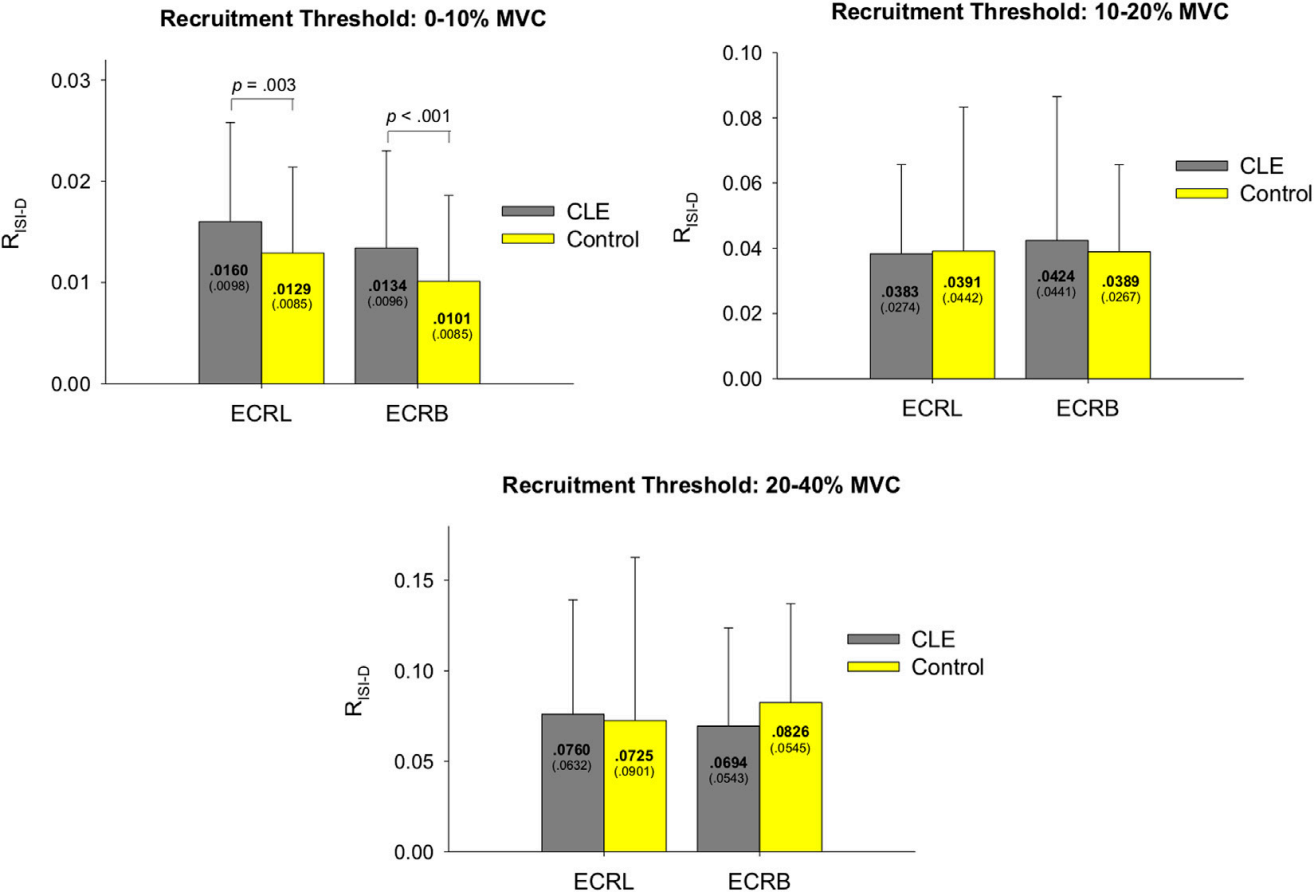
The contrast of the rate of progressive decrease in ISI (R_ISI-D_) of motor units with various recruitment thresholds in the ECRL and ECRB muscles between the patient and control groups. (CLE, chronic lateral epicondylitis).

The logarithmic linear model generally fits well for the decreasing trend of ISI of motor units during increasing force (Figure 3, right). For the patient group, the coefficient of determination (R^2^) for motor units with recruitment thresholds <10% MVC, 10%–20% MVC, and 20%–40% MVC were as follows: ECRL (0.550 ± 0.161, 0.586 ± 0.115, 0.638 ± 0.118) and ECRB (0.507 ± 0.206, 0.609 ± 0.219, 0.493 ± 0.211). For the control group, the coefficient of determination (R^2^) for motor units with recruitment thresholds <10% MVC, 10%–20% MVC, and 20%–40% MVC were as follows: ECRL (0.511 ± 0.162, 0.599 ± 0.111, 0.585 ± 0.149) and ECRB (0.472 ± 0.205, 0.584 ± 0.163, 0.551 ± 0.202). Figure 5 illustrates the rate of progressive decrease in ISI (R_ISI-D_) of motor units with various recruitment thresholds in the ECRL and ECRB muscles, comparing the patient and control groups. For motor units with recruitment thresholds <10% MVC, the R_ISI-D_ of the patient group was significantly higher than that of the healthy group (*p* < 0.005), regardless of the ECRL and ECRB muscles. However, there was no significant group difference in R_ISI-D_ for motor units with recruitment thresholds greater than 10% MVC (*p* > 0.05, *p* = 0.213–0.382).

## 4 Discussion

Along with a decline in MVC of wrist extension, this study demonstrated for the first time that the force gradation of patients with CLE was impaired during force increment rather than during force release. Functionally, the size and complexity of force fluctuations during force increment were significantly correlated to the MVC of wrist extension. The size of force fluctuations exhibited a marginally negative correlation with power grip strength and pain score. The deficits in precision control of increasing force were associated with a higher rate of progressive decrease in inter-spike interval of low-threshold motor units in the ECRB and ECRL muscles. In addition, motor unit action amplitude for patients with CLE was generally smaller than that of their healthy counterparts.

In this study, the force generation capacity of common wrist extensors was remarkably impaired in patients with CLE, as the MVC_WE_ of the patients was about 65.7% of that of the control group (Table 2). This finding was consistent with most previous work, which reported decreases in maximal wrist extension of 8%–33% (Heales et al., 2016). However, peak grip force did not differ between the two groups (Table 2), probably because grip force measurement could be confounded by wrist posture, coactivation of wrist antagonist pairs, equipment, shoulder position, and so on (Manickaraj et al., 2018). Hence, previous work considered that grip force deficit might not adequately account for clinical tests (Lindstroem et al., 2012; Pitts et al., 2021) or differ between the symptomatic and non-symptomatic limbs of patients with LE (Heales et al., 2016; Ucurum et al., 2019).

Given the greater task errors and the size of force fluctuations in the ramp-up phases (Table 3), patients with CLE exhibited a prominent deficit in force increment rather than in force release. This finding was somewhat unexpected because precise control of force release is more difficult than that of force increment (Park et al., 2016; Naik et al., 2011; Seo et al., 2009). Differential control for increasing and releasing force relate to recruitment and decruitment of motor units with central common drive at the gamma band (Naik et al., 2011; Park et al., 2016). By analogy with insignificant difference in releasing force characteristics (Table 3), supraspinal inhibitory influences on MUs for force release (Kamen and De Luca, 1989; Seo et al., 2009) were not severely undermined in the patients with CLE. Functionally, changes in the size (*r* = −0.660, *p* = 0.007) and complexity (*r* = 0.562, *p* = 0.029) of force fluctuations during force increment were negatively and positively correlated to MVC_WE_, respectively (Table 4). This finding indicated that those who exhibited poor force generation capacity tended to have greater variability during force increment, with less strategic richness to remedy tracking deviations (Hwang et al., 2017; Chen et al., 2018). That is, force fluctuation characteristics during increasing force nicely predict impairment of maximal wrist extension. Intriguingly, the force fluctuation characteristics (or force precision control) could not be well predicted by any known clinical questionnaires for LE (*p* > 0.05). Also, perceived pain seems not to be a potent factor in the inferior force gradation of patients with CLE (*p* = 0.076).

The deficits in increasing force observed in patients with CLE are associated with adaptive changes in motor units due to tendon damage. It is important to note that the mean amplitudes of the motor unit action potentials (MUAPs) in the ECRB and ECRL muscles of the patients were smaller compared to those of the healthy individuals (Table 5). This finding is consistent with the scarcity of active motor units with higher recruitment thresholds above 40% MVC, and the patient group showed a tendency to recruit low-threshold motor units during force increment (Figure 4). Our results are compatible with the findings of Calder et al. (2008), who reported degenerative changes characterized by smaller motor unit action potentials (MUAPs) in the ECRB muscle when using needle and decomposed surface electromyography (EMG) techniques. Corollary animal studies have shown that fast-twitch fibers have narrower and weaker Z disks, so the optimum length for tension is shorter for fast-twitch fibers than for slow-twitch fibers (Fridén and Lieber, 1998). Therefore, according to the sarcomere non-uniformity hypothesis, the sarcomeres of fast-twitch fibers are more susceptible to disruption when exposed to repetitive strains (Fridén and Lieber, 1998; Proske and Morgan, 2001). This vulnerability is particularly evident when the muscle is lengthened during contraction, as seen in activities like plyometric exercise (Macaluso et al., 2012; Isaacs et al., 2019). Functionally, the specific loss of larger motor units adversely affects the capacity for force generation and the ability to scale force increments in patients with CLE. The activation of motor units with higher recruitment thresholds is crucial for enhancing the efficiency of increasing force by combining the high twitch forces generated by those motor units.

In addition to motor unit recruitment, the firing rate of motor units plays a crucial role in force generation. In the context of the experimental pain model (Hodges et al., 2008; Poortvliet et al., 2015), it has been observed that perceived pain can inhibit motor units (Farina et al., 2008; Martinez-Valdes et al., 2021), particularly those with lower recruitment thresholds, pertaining to increase in variability of neural drive and decreases motor unit coherence at beta band to muscle (Yavuz et al., 2015). The pain-induced inhibition of low-threshold motor unit discharge, which exacerbates force fluctuations, can be compensated by recruiting additional high-threshold motor units. Nevertheless, it is important to note that the predictions made by the experimental pain model are based on observations from studies where healthy subjects were injected with hypertonic saline (Farina et al., 2004; Tucker et al., 2009; Martinez-Valdes et al., 2020). In the case of patients with CLE, the chronic degenerative changes in motor units, especially the selective damage to high-threshold motor units, hinder their ability to recruit these units as a compensatory mechanism. These patients rely on an alternative compensation strategy, which involves enhancing the acceleration rate of progressively shortening inter-spike intervals in the remaining low-threshold motor units to generate force (Figure 5). This compensatory rate coding may facilitate the fusion of twitch forces (Hwang et al., 2020), but force precision control of increasing force without ample recruitment of high-threshold motor units is still inferior compared to their healthy counterparts (Table 3).

Several methodological challenges merit concern. First, the patient participants were recruited from a single local hospital rather than multiple medical centers, which may confine the generalizability of the findings of this research. Next, the relatively small sample in this study may have caused sampling bias and unrepresentativeness of the patient groups, who had various pathological origins of CLE. Thirdly, the decomposition accuracy and number of identified MUs from surface EMG varied with individuals, which may have affected estimation of motor unit variables on an individual basis. Hence, this study was to compare motor unit variables using pooled data from all subjects with permutation test for statistical robustness. Despite the methodological constraints, our findings still provide novel insight into the MU pathophysiology and corresponding force scaling impairment of patients with CLE.

## 5 Conclusion

Conventional clinical questionnaires, including the visual pain scale, were not able to predict the impaired force precision control in CLE patients. In addition to a decline in force generation capacity, the impairment of force precision control was more prominent during force increment compared to force release. The force regulation impairment associated with a selective loss of larger motor units in CLE patients, characterized by a smaller proportion of motor units with higher recruitment thresholds and smaller motor unit action potentials. The compensatory mechanism involves an increase in the discharge rate acceleration of the remaining smaller motor units during force increment, which cannot be satisfactorily explained by the experimental pain model. These findings have significant implications for rehabilitation strategies in CLE patients, emphasizing the importance of training fine force control across various intensity ranges rather than solely focusing on maximal force strength training.

## Data Availability

The original contributions presented in the study are included in the article/Supplementary Material, further inquiries can be directed to the corresponding author.
